# Cross-sectional examination of metabolites and metabolic phenotypes in uremia

**DOI:** 10.1186/s12882-015-0100-y

**Published:** 2015-07-07

**Authors:** Sahir Kalim, Clary B. Clish, Joseph J. Deferio, Guillermo Ortiz, Alexander S. Moffet, Robert E. Gerszten, Ravi Thadhani, Eugene P. Rhee

**Affiliations:** 1 Division of Nephrology, Massachusetts General Hospital (MGH), 165 Cambridge Street, Suite 302, Boston, MA 02114 USA; 2 Broad Institute, Cambridge, MA USA; 3 Cardiology Division, MGH, Boston, MA USA; 4 Cardiovascular Research Center, MGH, Boston, MA USA

**Keywords:** Dialysis, Metabolism, Metabolomics, Uremic toxins

## Abstract

**Background:**

Although metabolomic approaches have begun to document numerous changes that arise in end stage renal disease (ESRD), how these alterations relate to established metabolic phenotypes in uremia is unknown.

**Methods:**

In 200 incident hemodialysis patients we used partial least squares discriminant analysis to identify which among 166 metabolites could best discriminate individuals with or without diabetes, and across tertiles of body mass index, serum albumin, total cholesterol, and systolic blood pressure.

**Results:**

Our data do not recapitulate metabolomic signatures of diabetes and obesity identified among individuals with normal renal function (*e.g.* elevations in branched chain and aromatic amino acids) and highlight several potential markers of diabetes status specific to ESRD, including xanthosine-5-phosphate and vanillylmandelic acid. Further, our data identify significant associations between elevated tryptophan and long-chain acylcarnitine levels and both decreased total cholesterol and systolic blood pressure in ESRD. Higher tryptophan levels were also associated with higher serum albumin levels, but this may reflect tryptophan’s significant albumin binding. Finally, an examination of the uremic retention solutes captured by our platform in relation to 24 clinical phenotypes provides a framework for investigating mechanisms of uremic toxicity.

**Conclusions:**

In sum, these studies leveraging metabolomic and metabolic phenotype data acquired in a well-characterized ESRD cohort demonstrate striking differences from metabolomics studies in the general population, and may provide clues to novel functional pathways in the ESRD population.

**Electronic supplementary material:**

The online version of this article (doi:10.1186/s12882-015-0100-y) contains supplementary material, which is available to authorized users.

## Background

End stage renal disease (ESRD) is characterized by various metabolic disturbances linked to adverse outcomes, but the nature of these associations is poorly understood or even counterintuitive [1]. For example, low serum albumin has consistently been linked to cardiovascular mortality in dialysis [2, 3], yet the association is not simply a result of inadequate nutrition, as intradialytic parenteral nutrition does not always improve survival in malnourished ESRD patients [4], and alternative pathways including inflammation are known to contribute [5]. Dyslipidemia is also common in ESRD, including atherogenic increases in carbamylated and oxidized-LDL cholesterol. Clinical trials, however, have failed to demonstrate a survival benefit with statin therapy in ESRD, despite substantial reductions in LDL cholesterol [6, 7]. Indeed, several studies have identified an inverse association between cholesterol levels and uremic cardiovascular risk [8, 9]. Similar paradoxical associations, sometimes described as examples of ‘reverse epidemiology’ in ESRD, have been noted with body mass index (BMI) and blood pressure [8, 10–12] and survival on hemodialysis.

Metabolomic approaches enable high resolution interrogation of metabolic phenotypes. For example, studies have begun to highlight specific metabolites associated with obesity and diabetes risk in the general population, particularly branched-chain and aromatic amino acids, but also short chain acylcarnitines, the glutamate/glutamine ratio, and bile acids [13–23]. Ultimately, these findings may lead to a more refined understanding of underlying disease mechanisms. Whether these and other associations identified in the burgeoning metabolomics literature extend to the ESRD population is unknown. While initial applications of metabolomics to nephrology research have generated a broad view of uremia [24] and the immediate effects of the hemodialysis procedure [25, 26], no studies have explicitly examined the cross-sectional relationship between uremic metabolite alterations and metabolic or other clinical phenotypes captured in a given ESRD study population.

We recently performed liquid chromatography-mass spectrometry (LC-MS) based metabolite profiling on plasma obtained from incident hemodialysis subjects in the Accelerated Mortality on Renal Replacement (ArMORR) cohort, and highlighted baseline levels of long-chain acylcarnitines as markers of future cardiovascular death in a longitudinal study [27]. Using this data set, we now leverage the rich clinical phenotyping available in ArMORR to examine cross-sectional associations between plasma metabolite levels and select metabolic phenotypes. Specifically, we sought to identify associations between metabolites and metabolic phenotype data in this well-characterized ESRD cohort, hypothesizing that these findings would differ from metabolomic associations derived from the general population. The aim of such work is to gain new insights into the functional metabolic pathways that underlie the poorly understood associations between clinical outcomes in ESRD and variables such as BMI, albumin, cholesterol, and blood pressure. Further, we examined the correlation between the subset of metabolites measured by our platform that are known uremic retention solutes and numerous clinical variables captured in ArMORR. Taken together, these results provide a new perspective on the metabolic disturbances that accompany and arise in ESRD.

## Methods

### Study population

The Accelerated Mortality on Renal Replacement (ArMORR) study is a prospective cohort study of 10,044 incident hemodialysis patients in any of 1,056 U.S. centers operated by Fresenius Medical Care North America between June 2004 and August 2005. Full details have been previously published [27–31]. All subjects underwent 1 year of follow-up except for those who died (15.2 %), voluntarily discontinued dialysis (5 %), underwent kidney transplantation (3 %), recovered renal function (4 %), or transferred to a dialysis unit outside the Fresenius system (12 %). Clinical data were prospectively collected by physicians at the point-of-care and included demographics, coexisting conditions, and routine laboratory studies. Plasma samples drawn at the beginning of a dialysis session within 14 days of initiation of outpatient hemodialysis, and that would otherwise have been discarded after routine clinical testing, were saved and stored in liquid nitrogen. We previously evaluated the associations between metabolite profile and mortality in the ArMORR cohort in a study of 100 randomly selected patients who died within the first year of dialysis from a cardiovascular cause, and 100 patients who were alive 1 year after starting dialysis, frequency matched for age, sex, and race [27]. We included this entire group of individuals in the current study. This study was approved by the Massachusetts General Hospital IRB, which waived the need for informed consent because all personal identifiers were removed from the blood samples and clinical data before transfer to the investigators.

### Metabolite profiling

We applied two distinct LC-MS based methods to distinct plasma aliquots for each study subject. Amino acids, amino acid derivatives, urea cycle intermediates, nucleotides and other positively charged polar metabolites were profiled as previously described [14]. Briefly, 10 μL of plasma were extracted with 90 μL of 74.9:24.9:0.2 vol/vol/vol acetonitrile/methanol/formic acid containing valine-d8 (Sigma-Aldrich; St Louis, MO). After centrifugation, supernatants underwent hydrophobic interaction chromatography using a 150 × 2.1 mm Atlantis hydrophobic interaction chromatography column (Waters, Milford, MA), and MS data were acquired on a 4000 QTRAP triple quadrupole mass spectrometer (AB SCIEX, Foster City, CA) using ESI and MRM in the positive ion mode. Organic acids, sugars, bile acids, and other negatively charged polar metabolites were profiled as previously described [32]. Briefly, 30 μL of plasma were extracted with the addition of four volumes of 80:20 vol/vol methanol/water containing isotope-labeled inosine-15 N4, supernatants underwent chromatography on a 150 × 2.0 mm Luna NH2 column (Phenomenex; Torrance, CA), and MS data were acquired using a 5500 QTRAP triple quadrupole mass spectrometer (AB SCIEX; Foster City, CA) using ESI and MRM in the negative ion mode.

### Statistical analyses

We first examined the association between plasma metabolites and pre-selected metabolic phenotypes: diabetes status, BMI, serum albumin, total cholesterol, and systolic blood pressure (SBP). We focused on these phenotypes because of their strong links to metabolism and their established, but incompletely understood, associations with ESRD mortality [33–39]. To investigate differences in metabolite profiles across these phenotypes, continuous outcomes measures (BMI, albumin, total cholesterol, and SBP) were divided into tertiles. Diabetes status was classified as “yes” or “no”.

Because of the number of metabolites measured by our LC-MS platform (166 total, Additional file 1: Table S1), many with significant inter-correlations, we performed partial least squares discriminant analysis (PLS-DA) to visualize the linear components that discriminate individuals across the categories (tertiles or class) of metabolic phenotypes. PLS-DA was performed on log transformed and auto-scaled (mean-centered and divided by the standard deviation of each metabolite) data using MetaboAnalyst 2.0 [40, 41]. This program performs PLS regression using the *plsr* function provided by the pls package in R, and classification and cross validation using the corresponding wrapper function offered by the caret package. Variable Importance in Projection (VIP) scores generated by this program estimate the importance of each variable in the projection used within the PLS model. A variable with a VIP score greater than 1 can be considered important in a given model. The metabolites with the highest VIP scores were further analyzed by comparing their levels across classes using Mann-U-Whitney and Kruskal-Wallis tests as appropriate.

Finally, heatmaps were created to represent Pearson correlation (r) and *P*-value matrices between available clinical variables in the cohort and the subset of metabolites measured by our platform that have previously been described as uremic retention solutes (http://www.uremic-toxins.org) [27, 42–45].

To account for multiple testing, we used a Bonferroni corrected significance threshold of *P* < 0.0003 (0.05/166) for comparisons of individual metabolites across phenotype class. For the correlation matrices, we used an adjusted significance threshold of *P* < 0.00007 (0.05/[29 metabolites* 24 clinical variables]). A sensitivity analysis was performed to gauge the influence of case–control status (*i.e.* whether or not a given individual died of a cardiovascular cause within one year of starting dialysis) on the associations described herein. Stratified analysis by case status did not alter the statistical significance of the models described. Therefore, we did not stratify models by mortality status and analyzed the entire group of 200 individuals together as a cohort. All analyses were performed using SAS software version 9.1.3 (SAS Institute) and MetaboAnalyst 2.0 software (www.metaboanalyst.ca).

## Results and discussion

### Cohort characteristics

As shown in Table 1, the mean age of the study population was 69.5 years and 69 % of subjects were white. There was an equal representation of males and females and nearly half of the individuals had a history of diabetes or had diabetes listed as their cause of ESRD (49 %). The mean BMI was 26.5 kg/m^2^ (SD ±7.6 kg/m^2^) and the mean SBP was 144 mmHg (±27 mmHg). A minority of individuals reported a lipid disorder (12 %) and the median total cholesterol level was 162 mg/dL (quartile1-quartile3, 127–188 mg/dL). The median serum albumin level was 3.6 g/dl (3.2-3.8 g/dL).

**Table 1:** Baseline characteristics of the study sample

Variable	Total *n* = 200
Age, years	69.5 ± 13.6
Male	53 % (106)
Race	
White	69 % (138)
Black	24 % (48)
Other	7 % (14)
Coexisting conditions	
Coronary artery disease	18 % (36)
Lipid disorders	12 % (23)
Congestive heart failure	18 % (35)
Cause of end-stage renal disease	
Diabetes^a^	49 % (97)
Hypertensive renal disease	38 % (75)
Glomerulonephropathy	4 % (7)
Vascular access	
Fistula	24 % (47)
Graft	13 % (25)
Catheter	59 % (118)
Body Mass Index (kg/m^2^)	26.5 ± 7.6
Systolic blood pressure (mmHg)	144.4 ± 27.3
Diastolic blood pressure (mmHg)	72.5 ± 18.6
Urea reduction ratio	68.6 ± 10.3
Laboratory data	
N-terminal pro-BNP (ng/L)	8029 (3,757 – 21,404)
Troponin T (μg/L)	0.14 (0.04 – 0.13)
Total cholesterol (mg/dL)	162 (127 – 188)
LDL (mg/dL)	83 (63 – 103)
HDL (mg/dL)	41 (32 – 47)
Triglycerides (mg/dL)	157 (93 – 199)
Creatinine (mg/dL)	5.7 (4.0 – 7.0)
White blood cell (10^9^/L)	8.3 (6.2 – 9.8)
Hemoglobin (g/dL)	10.4 (9.5 – 11.2)
Albumin (g/dL)	3.5 (3.2 – 3.8)
Ferritin (ng/mL)	285.7 (81.5 – 346.0)
Transferrin saturation (%)	20.2 (13.0 – 24.0)
Phosphorus (mg/dL)	4.5 (3.4 – 5.4)
Parathyroid hormone (pg/mL)	268.6 (124.0 – 235.0)

Categorical data are percentages (counts). Counts may not equal total n due to missing data. Clinical measures are means ± SD. Laboratory values are median (quartile 1 – quartile 3)

BNP, brain natriuretic peptide

^a^Includes all patients with diabetes

### Examination of metabolite profiles and select metabolic phenotypes

**Fig. 1:**
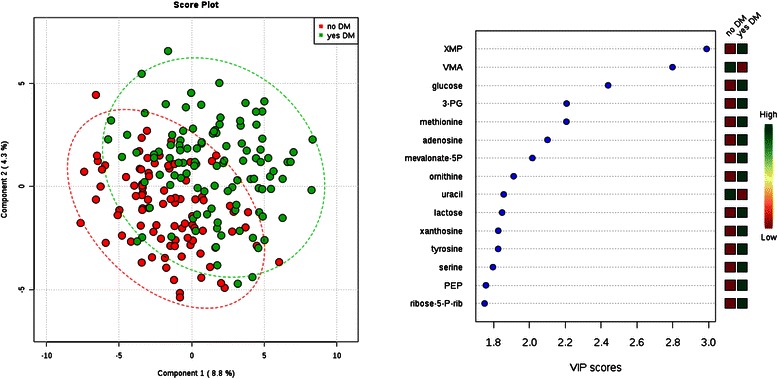
Comparison of metabolite profiles and diabetes status. Study subjects were grouped by diabetes status (yes/no). Left: Partial least squares discriminant analysis (PLS-DA) score plot for the study population separated by phenotype class. Oval outlines denote 95 % confidence intervals. Right: The variable importance in projection (VIP) scores for the 15 metabolites with the greatest score. The colored boxes on the right indicate the direction of metabolite alterations across phenotype class. Abbreviations: XMP, Xanthosine-5-phosphate; VMA, vanillylmandelic acid; 3-PG, 3-phosphoglycerate; mevalonate-5P, mevalonate-5-phosphate; PEP, phosphoenolpyruvate; ribose-5P, ribose-5-phosphate and ribulose-5-phosphate

**Fig. 2:**
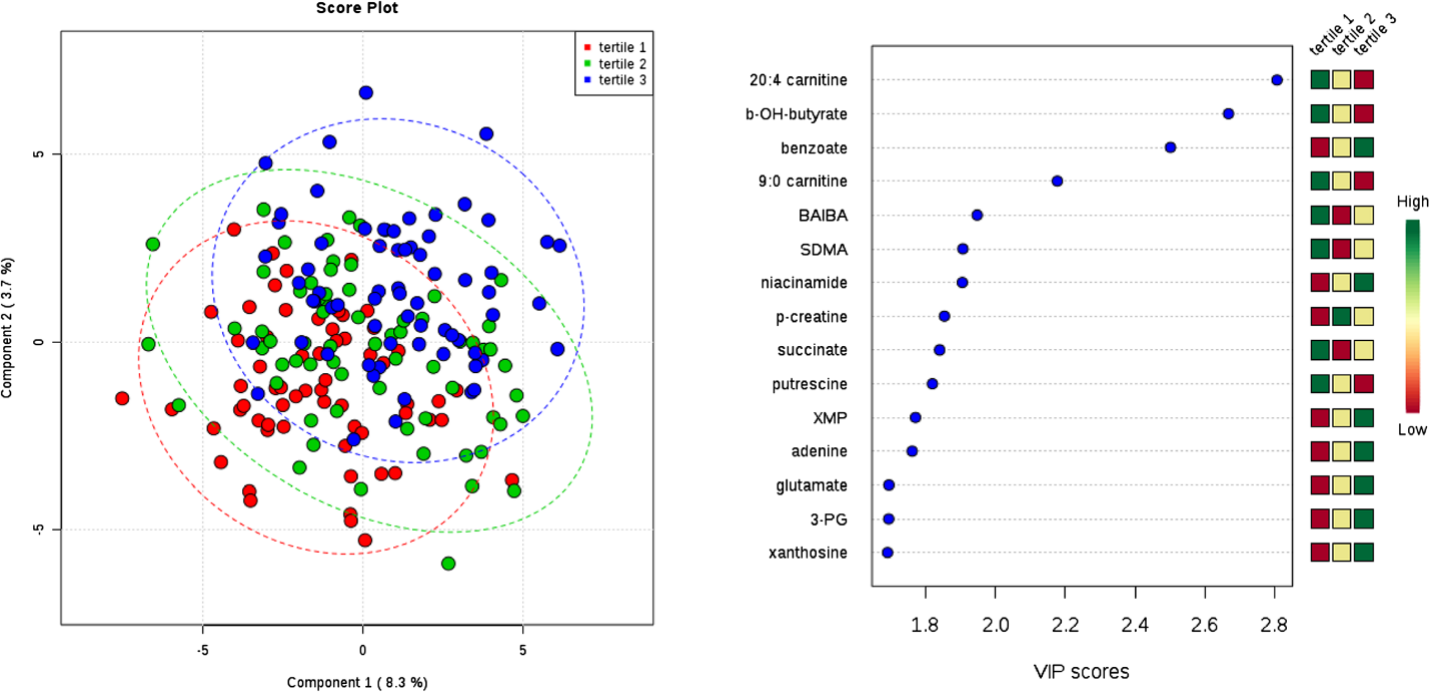
Comparison of metabolite profiles across tertile of body mass index (BMI). Study subjects were grouped by BMI tertile (tertile 1 = lowest BMI). Left: Partial least squares discriminant analysis (PLS-DA) score plot for the study population separated by phenotype class. Oval outlines denote 95 % confidence intervals. Right: The variable importance in projection (VIP) scores for the 15 metabolites with the greatest score. The colored boxes on the right indicate the direction of metabolite alterations across phenotype tertile. Abbreviations: BAIBA, β-aminoisobutyric acid; SDMA, symmetric dimethylarginine; 3-PG, 3-phosphoglycerate; b-OH-butyrate, β-hydroxybutryate; p-creatine, phosphocreatine; XMP, Xanthosine-5-phosphate

**Fig. 3:**
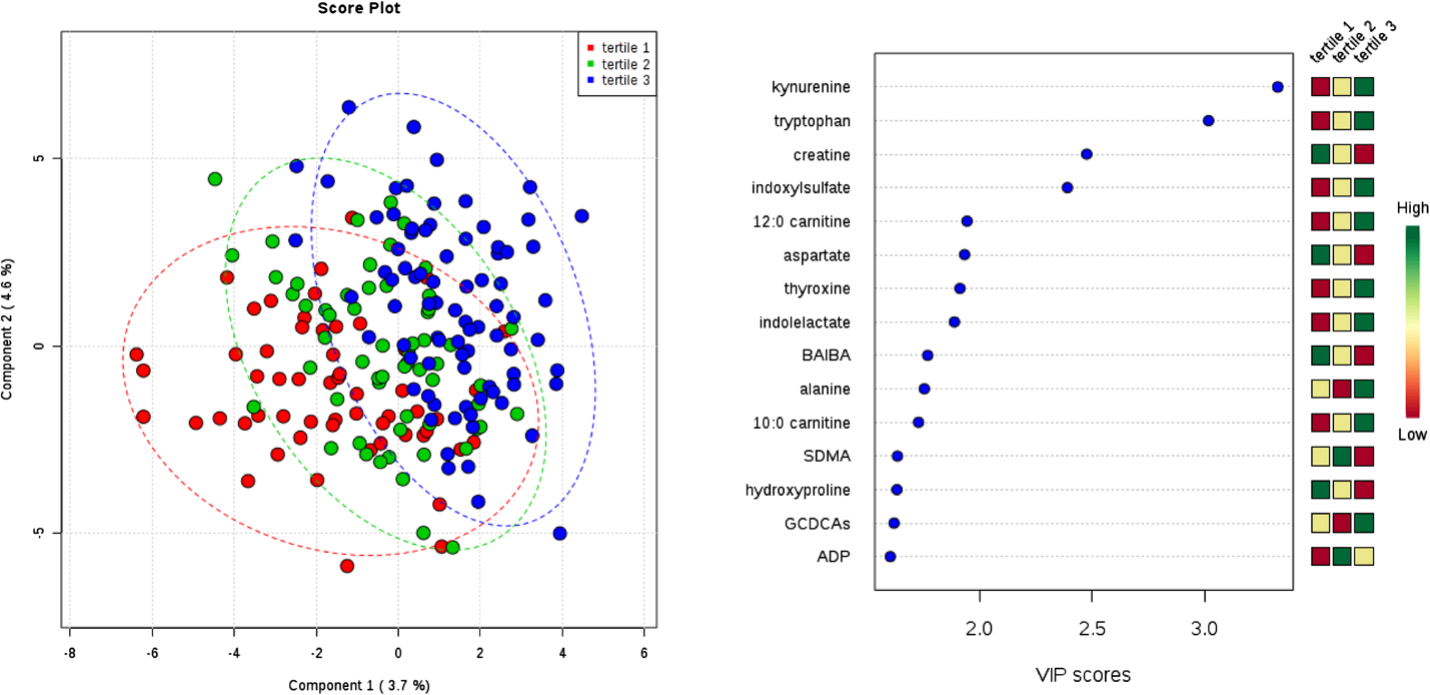
Comparison of metabolite profiles across tertile of serum albumin. Study subjects were grouped by albumin tertile (tertile 1 = lowest). Left: Partial least squares discriminant analysis (PLS-DA) score plot for the study population separated by phenotype class. Oval outlines denote 95 % confidence intervals. Right: The variable importance in projection (VIP) scores for the 15 metabolites with the greatest score. The colored boxes on the right indicate the direction of metabolite alterations across phenotype tertile. Abbreviations: BAIBA, β-aminoisobutyric acid; SDMA, symmetric dimethylarginine; GCDCAs, glycodeoxycholate and glycochenodeoxycholate; ADP, adenosine diphosphate

**Fig. 4:**
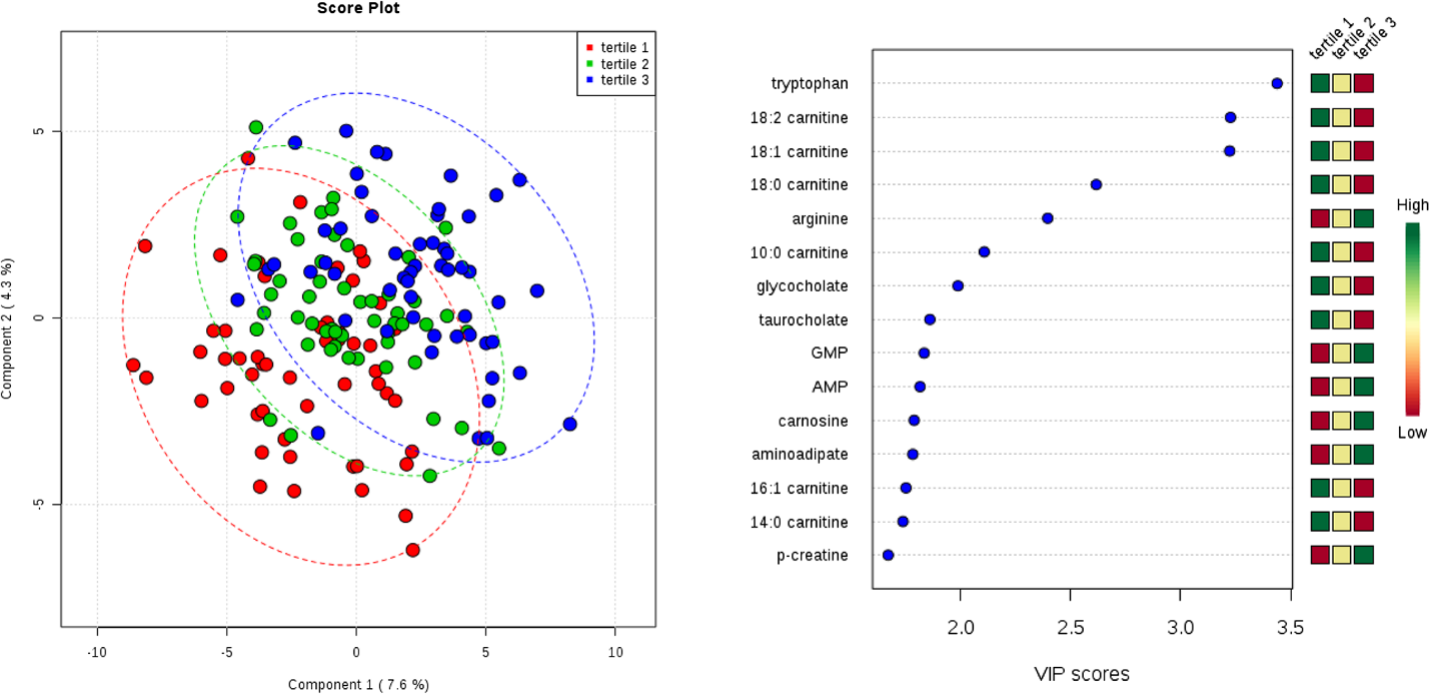
Comparison of metabolite profiles across tertile of total cholesterol. Study subjects were grouped by cholesterol tertile (tertile 1 = lowest). Left: Partial least squares discriminant analysis (PLS-DA) score plot for the study population separated by phenotype class. Oval outlines denote 95 % confidence intervals. Right: The variable importance in projection (VIP) scores for the 15 metabolites with the greatest score. The colored boxes on the right indicate the direction of metabolite alterations across phenotype tertile. Abbreviations: GMP, guanosine monophosphate; AMP, adenosine monophosphate; p-creatine, phosphocreatine

**Fig. 5:**
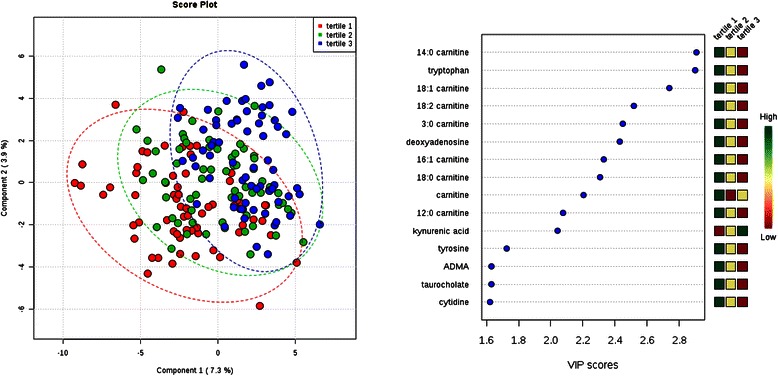
Comparison of metabolite profiles across tertile of systolic blood pressure. Study subjects were grouped by systolic blood pressure tertile (tertile 1 = lowest). Left: Partial least squares discriminant analysis (PLS-DA) score plot for the study population separated by phenotype class. Oval outlines denote 95 % confidence intervals. Right: The variable importance in projection (VIP) scores for the 15 metabolites with the greatest score. The colored boxes on the right indicate the direction of metabolite alterations across phenotype tertile. Abbreviations: ADMA, asymmetric dimethylarginine

The PLS-DA approach allowed us to visualize and extract the metabolites that best separated individuals (Figs. 1,2,3,4 and 5; left panels) across phenotype tertile or class (Table 2). Because four of the clinical phenotypes we studied (BMI, serum albumin, total cholesterol, and SBP) are continuous measures, we recognize that tertile cut-offs do not demarcate distinct physiologic or pathophysiologic processes. Thus, plot overlap across tertiles was expected. Variable importance in projection (VIP) provided a score for each metabolite, ranking the metabolites according to their predictive power in the PLS model; the fifteen metabolites with the highest VIP scores for each plot are shown in Figs. 1, 2, 3, 4 and 5 (right panels), and the levels of these metabolites across tertiles (or class) of the phenotypes with corresponding test statistics are shown in Tables 3-7.

**Table 2:** Primary metabolic phenotypes by tertiles

	Tertile	Measure
Body mass index (kg/m^2)^	1	20.2 ± 3.9
	2	25.6 ± 1.3
	3	34.2 ± 7.6
Albumin (g/dl)	1	3.1 (2.8, 3.2)
	2	3.6 (3.4, 3.7)
	3	4.0 (3.8, 4.2)
Cholesterol (mg/dl)	1	116.0 (106.0-126.0)
	2	154.0 (146.0-163.0)
	3	209.5 (188.0-236.0)
Systolic blood pressure (mmHg)	1	116.1 ± 10.0
	2	142.2 ± 7.0
	3	174.6 ± 20.2

Clinical measures are mean ± SD and laboratory values are median (quartile 1 to quartile 3)

Total *n* = 200

**Table 3:** Cross sectional analysis of diabetes status and select metabolites

	no DM	DM	*P*
XMP	13 585 (8 386, 29 579)	62 101 (14 261, 158 938)	5.0E-7*
VMA	173 153 (107 165, 279 690)	104 186 (67 902, 164 683)	1.5E-6*
Glucose	2 935 (1 046, 5 294)	6 245 (2 777, 9 807)	3.1E-6*
3-PG	2 475 150 (1 882 036, 333 2264)	3 767 372 (2 250 378, 5 598 373)	4.9E-5*
Methionine	195 026 (152 633, 239 841)	243 230 (195 219, 283 910)	4.5E-5*
Adenosine	1 942 (1 463, 3 457)	3 078 (1 706, 5 137)	8.6E-4
mevalonate-5P	192 139 (136 927, 291 930)	292 904 (165 128, 433 871)	2.8E-4*
ornithine	1 385 224 (1 083 311, 1 721 772)	1 657 626 (1 351 270, 1 913 549)	3.2E-4*
uracil	6 888 567 (4 490 348, 9 622 913)	4 784 283 (3 740 917, 6 592 407)	5.7E-4
lactose	3 140 699 (2 178 153, 4 835 219)	4 519 336 (2 900 447, 6 650 384)	3.8E-4
xanthosine	10 839 (7 518, 22 311)	22 007 (8 845, 36 474)	1.0E-3
tyrosine	222 479 (174 354, 277 562)	247 875 (210 621, 309 004)	2.2E-3
serine	352 671 (280 146, 415 607)	381 134 (319 491, 476 035)	4.6E-3
PEP	312 003 (189 719, 522 172)	462 016 (257 705, 925 860)	1.3E-3
ribose-5-P-rib	144 443 (104 797, 208 719)	180 078 (116 088, 322 799)	8.7E-3

Values are median peak area for the metabolites (quartile 1, quartile 3)

*P-value significant at the Bonferroni adjusted level of 3.0 × 10−4

Values are median peak area for the metabolites (quartile 1, quartile 3)*P-value significant at the Bonferroni adjusted level of 3.0 × 10−4

For individuals with or without diabetes, glucose was one of the strongest discriminating metabolites, with higher levels among the individuals with diabetes (Fig. 1). Xanthosine-5-phosphate (XMP) and vanillylmandelic acid (VMA), however, had even higher VIP scores than glucose, with XMP levels higher and VMA levels lower among the individuals with diabetes (*P* = 5.0 **×** 10^−7^ and 1.5 **×** 10^−6^ for XMP and VMA, respectively). Other significant differences included higher levels of 3-phosphoglyceric acid (*P* = 4.9 **×** 10^−5^), methionine (*P* = 4.5 **×** 10^−5^), mevalonate-5-phosphate (*P* = 2.8 **×** 10^−4^), and ornithine (*P* = 3.2 **×** 10^−4^) among individuals with diabetes. Tyrosine, an aromatic amino acid, had a nominal association with diabetes status (*P* = 1.0 **×** 10^−3^). There were no significant associations between tyrosine levels, or any aromatic or branched-chain amino acid levels, with BMI (Table 4). In fact, none of these metabolites had even a nominal association with BMI in univariate comparisons (data not shown). Overall, the ability of metabolite profiles to discriminate individuals across tertiles of BMI was poor (Fig. 2). Among the top metabolites by VIP score, only 20:4 carnitine (arachidonyl carnitine), β-hydroxybutyrate, and benzoate had nominal *P*-values in univariate analysis (Table 4).

**Table 4:** Cross sectional analysis of BMI tertiles and select metabolites

	1	2	3	*P*
20:4 carnitine	296 (246, 378)	263 (216, 324)	253 (207, 292)	7.8E-3
b-OH-butyrate	508 950 (249 015, 1 818 943)	395 440 (234 716, 895 354)	343 616 (172 233, 717 989)	2.9E-2
benzoate	15 216 (11 526, 24 891)	18 382 (12 187, 28 788)	21 271 (14 892, 34 600)	4.1E-2
9:0 carnitine	17 878 (11 801, 23 836)	16 514 (9 560, 24 702)	13 239 (9 472, 20 371)	5.7E-2
BAIBA	49654 (26452, 107799)	36205 (24704, 57849)	38844 (23864, 74057)	1.3E-1
SDMA	942527 (769351, 1098799)	852551 (750082, 970184)	826704 (729114, 1062038)	7.4E-2
niacinamide	209151 (170065, 248736)	214718 (175857, 281191)	246744 (187545, 297341)	6.8E-2
p-creatine	42402 (31941, 51249)	47705 (39559, 61036)	48786 (37472, 57166)	4.0E-2
succinate	17 776 284 (15 961 017, 22 206 915)	15 637 602 (12 262 309, 19 434 811)	16 309 449 (12 852 577, 19 680 091)	6.1E-3
putrescine	68559 (44891, 109979)	54285 (18371, 87225)	53 504 (12105, 93032)	1.3E-1
XMP	21 819 (11 029, 53 381)	20 694 (8 856, 143 900)	37 771 (12 370, 121 441)	1.7E-1
adenine	90 351 (61 426, 165 720)	109 819 (79 107, 201 534)	116 489 (76 249, 194 627)	1.1E-1
glutamate	870 295 (691 881, 1 109 208)	992 968 (715 437, 1 375 593)	1 031 076 (714 204, 1 443 438)	1.2E-1
3-PG	2 824 102 (1 855 508, 4 336 931)	2 788 537 (1 930 889, 5 190 633)	3 294 065 (2 358 954, 5 383 792)	9.2E-2
xanthosine	12 923 (7 425, 24 088)	10 883 (7 604, 33 249)	19 991 (8 413, 30 597)	1.7E-1

Values are median peak area for the metabolites (quartile 1, quartile 3)

*P-value significant at the Bonferroni adjusted level of 3.0 × 10−4

Values are median peak area for the metabolites (quartile 1, quartile 3)*P-value significant at the Bonferroni adjusted level of 3.0 × 10−4

**Table 5:** Cross sectional analysis of albumin tertiles and select metabolites

	1	2	3	*P*
kynurenine	425 998 (298 054, 535 460)	478 927 (395 468, 596 735)	552 251 (433 248, 679 481)	1.8E-4*
tryptophan	273 066 (223 467, 413 194)	315 338 (255 914, 423 927)	393 551 (301 885, 501 066)	2.5E-4*
creatine	2 157 292 (1 397 576, 3 355 087)	1 907 461 (1 145 455, 2 791 140)	1 570 693 (1 182 023, 2 037 120)	4.9E-3
indoxylsulfate	42 510 713 (24 175 943, 58 375 318)	55 564 881 (34 527 474, 67 400 369)	57 398 073 (33 359 446, 80 148 935)	1.3E-2
12:0 carnitine	28 261 (18 195, 40 622)	30 496 (18 540, 44 940)	36 915 (24 245, 50 553)	2.7E-2
aspartate	36 448 (25 083, 49 840)	34 626 (24 685, 48 518)	26 533 (20 120, 36 067)	1.9E-3
thyroxine	2 332 (1 929, 2 738)	2 523 (1 879, 2 916)	2 712 (2 115, 3 244)	1.8E-2
indolelactate	12 722 (8 573, 19 334)	15 627 (11 171, 22 311)	16 269 (11 922, 22 700)	3.6E-2
BAIBA	46 979 (27 423, 106 593)	42 313 (24 146, 80 713)	34 051 (23 583, 57 272)	1.0E-1
alanine	1 280 822 (985 715, 1 604 978)	1 249 845 (1 028 428, 1 494 869)	1 337 859 (1 117 636, 1 758 661)	6.9E-2
10:0 carnitine	33 566 (21 210, 61 857)	48 413 (25 775, 68 324)	47 884 (31 469, 72 628)	5.8E-2
SDMA	896 522 (765 176, 1 090 288)	925 219 (772 578, 1 063 241)	824 248 (730 601, 974 498)	5.2E-2
hydroxyproline	235 578 (162 155, 374 510)	203 966 (149 720, 306 721)	198 496 (150 564, 259 207)	1.5E-1
GCDCAs	487 186 (203 751, 773 938)	421 065 (248 114, 748 019)	715 307 (356 712, 1 064 255)	1.4E-2
ADP	280 998 (155 075, 610 214)	539 330 (278 012, 877 605)	455 070 (164 681, 1 239 020)	1.2E-2

Values are median peak area for the metabolites (quartile 1, quartile 3)

*P-value significant at the Bonferroni adjusted level of 3.0 × 10−4

Values are median peak area for the metabolites (quartile 1, quartile 3)*P-value significant at the Bonferroni adjusted level of 3.0 × 10−4

Thus, our data from an ESRD population do not recapitulate observations derived in the general population linking diabetes and obesity with elevations in branched chain and aromatic amino acids (except for the nominal association with tyrosine noted above), short chain acylcarnitines, the glutamate/glutamine ratio, or bile acids [2]. One potential explanation is that kidney function (and dysfunction) has a direct effect on select metabolites. The interaction between kidney function and amino acid metabolism, in particular, has been closely examined. For example, the kidney is known to make a substantial contribution to whole body tyrosine appearance via intra-organ phenylalanine hydroxylation [46]. Further, the metabolic acidosis that results from renal failure leads to increased leucine oxidation [47], and indeed, generalized muscle catabolism. The significant associations we did identify with diabetes status in ESRD, *e.g.* XMP (a purine breakdown product) and VMA (a catecholamine metabolite), have not been reported in the general population. To what extent these alterations reflect distinct pathophysiologic processes in uremia warrant further investigation.

For individuals in different tertiles of serum albumin, PLS-DA demonstrated moderate separation across groups (Fig. 3). Tryptophan and its downstream metabolite kynurenine were significantly higher in individuals with higher serum albumin levels (*P* = 1.8 × 10^−4^ and 2.5 × 10^−4^, respectively). Two additional tryptophan metabolites, indoxyl sulfate and indole lactate, had a trend for higher levels among individuals with higher serum albumin levels. Given recent observations that ascribe a functional role for tryptophan metabolism, specifically through indoleamine-2, 3-dioxygenase (IDO), in modulating the immune system and vascular tone [48, 49], it is an appealing link between metabolism, nutritional status, inflammation, and cardiovascular risk in ESRD. However, these data should be interpreted with caution. Because tryptophan and its catabolites are hydrophobic, their positive correlation with albumin may reflect their significant protein-binding [50] – our LC-MS method measures total, not free, plasma metabolite levels. A similar mechanism may underlie the trend for association between thyroxine and albumin levels. By contrast, creatine, the metabolite with the third highest VIP score, had a trend for higher levels among individuals with lower serum albumin levels. In a study that examined spent media from cultured muscle cells treated with mitochondrial respiratory chain inhibitors, as well as plasma obtained from individuals with respiratory chain diseases, creatine levels were reproducibly elevated [51]. In cell culture, extracellular creatine was inversely correlated with the intracellular phosphocreatine/creatine ratio, suggesting that elevated plasma creatine may signal a low energetic state in tissues using the creatine phosphate shuttle. Interestingly, elevated plasma creatine and decreased plasma phosphocreatine levels both have a nominal association with increased 1-year cardiovascular mortality among incident dialysis patients in ArMORR [27]. These observations raise the possibility that impaired mitochondrial respiration in muscle is linked to the pathogenesis of hypoalbuminemia in dialysis.

For individuals across tertiles of total cholesterol and SBP, PLS-DA again demonstrated moderate separation across tertiles (Figs. 4 and 5). For both analyses, long-chain acylcarnitines were among the metabolites with the highest VIP scores, with 18:2 carnitine (linoleylcarnitine, *P* = 9.0 × 10^−7^), 18:1 carnitine (oleoylcarnitine, *P* = 6.0 × 10^−7^), and 18:0 carnitine (stearoylcarnitine, *P* = 7.7 × 10^−5^) all significantly higher in individuals with lower total cholesterol levels (Table 6,) and linoleylcarnitine (*P* = 5.5 × 10^−5^) and oleoylcarnitine (*P* = 8.7 × 10^−6^) significantly higher in individuals with lower SBP (Table 7); select short-chain and medium-chain acylcarnitines were also significantly elevated among individuals with lower SBP. Like albumin, low cholesterol and blood pressure are established predictors of increased mortality in ESRD [8, 9, 11, 12]. These findings are consistent with our observation that long-chain acylcarnitines are associated with 1-year cardiovascular mortality in ESRD [27], and motivate further interest in long-chain acylcarnitines as markers, or even mediators, of altered lipid metabolism and cardiovascular function in uremia. Tryptophan, one of the strongest discriminators of albumin tertile, was also a top hit in regards to total cholesterol and SBP, with higher tryptophan significantly associated with lower levels for both. Finally, higher arginine levels were significantly associated with higher total cholesterol and higher deoxyadenosine levels were significantly associated with lower SBP.

**Table 6:** Cross sectional analysis of total cholesterol tertiles and select metabolites

	1	2	3	*P*
tryptophan	440 068 (345 130, 586637)	323192 (257580, 429806)	274624 (223120, 324798)	1.7E-9*
18:2 carnitine	60 494 (38 250, 107 981)	49 737 (27 163, 73 344)	32 084 (19 167, 53 863)	9.0E-7*
18:1 carnitine	88 418 (58 090, 144 102)	69 442 (40 568, 103 682)	47 134 (33 127, 74 763)	6.0E-7*
18:0 carnitine	12 789 (10 118, 18 240)	10 879 (7 872, 14 622)	8 466 (6 103, 12 433)	7.7E-5*
arginine	175 576 (136 362, 259 524)	187 566 (132 389, 324 585)	321 420 (216 851, 491 848)	9.6E-6*
10:0 carnitine	52 498 (28 731, 92 694)	40 556 (26 190, 67 085)	34 244 (20 094, 52 420)	7.0E-3
glycocholate	193 580 (60 425, 672 001)	105 938 (61 765, 208 013)	76 985 (42 135, 222 324)	5.6E-3
taurocholate	128 124 (30 180, 379 765)	91 894 (35 067, 188 948)	47 535 (24 706, 128 025)	1.9E-2
GMP	640 409 (341 616, 884 723)	544 860 (409 493, 969 998)	831 864 (565 936, 1 246 583)	8.9E-3
AMP	2 898 935 (2 275 762, 3 795 325)	3 153 490 (2 679 559, 4 251 281)	3 708 724 (3 046 005, 4 670 016)	1.6E-3
carnosine	23 811 (15 639, 77 524)	34 287 (14 160, 119 781)	64 617 (29 846, 195 284)	6.5E-3
aminoadipate	139 402 (119 264, 184 086)	167 760 (130 029, 210 530)	186 821 (144 705, 242 940)	3.3E-3
16:1 carnitine	68 049 (46 259, 99 787)	65 354 (35 653, 77 665)	49 527 (39 008, 71 026)	2.1E-2
14:0 carnitine	9 893 (6 819, 17 108)	8 969 (5 659, 12 834)	7 848 (5 373, 10 960)	3.1E-2
p-creatine	42 102 (30 390, 51 406)	46 713 (36 982, 55 510)	50 600 (37 472, 70 045)	1.8E-2

Values are median peak area for the metabolites (quartile 1, quartile 3)

*P-value significant at the Bonferroni adjusted level of 3.0 × 10−4

Values are median peak area for the metabolites (quartile 1, quartile 3)*P-value significant at the Bonferroni adjusted level of 3.0 × 10−4

**Table 7:** Cross sectional analysis of systolic blood pressure tertiles and select metabolites

	1	2	3	*P*
14:0 carnitine	10 549 (7 384, 17 510)	7 855 (6 040, 11 626)	6 824 (4 844, 9 486)	9.0E-6*
tryptophan	433 424 (297 696, 573 865)	366 630 (261 744, 444 478)	285 101 (229 380, 350 075)	1.2E-6*
18:1 carnitine	86 406 (56 919, 127 989)	70 273 (47 764, 105 108)	52 275 (32 871, 76 097)	8.7E-6*
18:2 carnitine	55 715 (35 972, 84 528)	44 392 (31 334, 69 607)	30 733 (21 812, 54 084)	5.5E-5*
3:0 carnitine	770 205 (556 166, 976 463)	604 794 (378 770, 724 282)	549 619 (381 463, 708 095)	5.4E-5*
deoxyadenosine	882 (605, 1 330)	831 (455, 1 011)	571 (441, 787)	7.5E-5*
16:1 carnitine	70 042 (50 052, 97 483)	53 904 (39 997, 77 945)	47 577 (36 257, 68 016)	4.2E-4
18:0 carnitine	12 434 (8 777, 17 403)	10 456 (7 790, 15 356)	8 806 (6 509, 12 276)	4.2E-4
carnitine	4 940 542 (4 375 772, 5 974 275)	4 311 636 (3 616 956, 4 936 736)	4 279 513 (3 743 960, 4 756 471)	2.1E-5*
12:0 carnitine	39 844 (22 621, 61 584)	33 433 (22 258, 39 850)	27 094 (17 488, 43 033)	8.5E-3
kynurenic acid	63 185 (35 805, 129 532)	86 131 (60 982, 133 796)	116 334 (67 598, 169 586)	9.4E-4
tyrosine	255 780 (216 337, 319 582)	231 195 (200 964, 273 325)	223 332 (177 881, 270 817)	8.4E-3
ADMA	309 751 (281 198, 370 574)	290 947 (261 258, 344 552)	284 594 (248 154, 328 619)	1.1E-2
taurocholate	103 888 (39 457, 284 746)	65 463 (26 657, 196 110)	59 392 (25 323, 157 090)	1.8E-2
cytidine	60 033 (36 540, 80 779)	51 105 (32 838, 73 636)	44 821 (26 784, 61 200)	2.1E-2

Values are median peak area for the metabolites (quartile 1, quartile 3)

*P-value significant at the Bonferroni adjusted level of 3.0 × 10^−4^

### Relation of uremic solutes with twenty-four clinical phenotypes

**Fig. 6:**
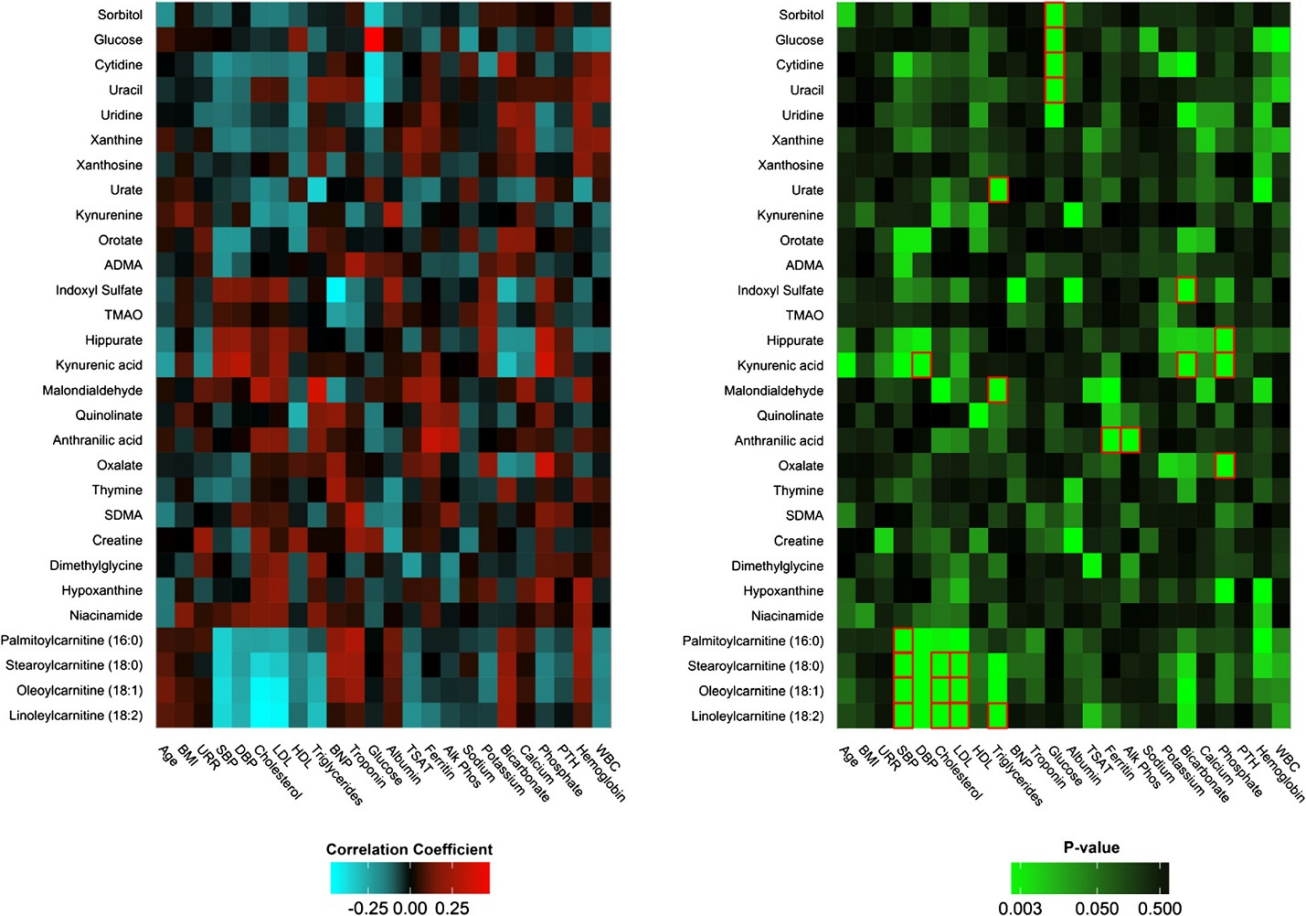
Relation of uremic solutes with 24 clinical phenotypes in the ArMORR cohort. Correlation coefficient (r, left) and *P* values (right) generated from Pearson correlation analyses of metabolites and available clinical variables in the ArMORR cohort. Squares outlined in red on the *P* value matrix represent values significant at the Bonferroni adjusted level of *P <* 7.4 × 10^−5^. Abbreviations: ADMA, asymmetric dimethylarginine; TMAO, trimethylamine-N-oxide; SDMA, symmetric dimethylarginine; BMI, body mass index; URR, urea reduction ratio; SBP, systolic blood pressure; DBP, diastolic blood pressure; LDL, low density lipoprotein; HDL, high density lipoprotein; BNP, b-type natriuretic peptide, TSAT, transferrin saturation; Alk Phos, alkaline phosphatase; PTH, parathyroid hormone; WBC, white blood cell

To examine the specificity of the long-chain acylcarnitine-phenotype associations described above, we looked more broadly at the correlation between all of the metabolites captured on our platform that are uremic retention solutes and 24 phenotypes captured in ArMORR. Correlations between previously reported uremic retention solutes measured by our LC-MS platform, including long-chain acylcarnitines, and a broad array of clinical phenotypes measured in ArMORR are shown in Fig. 6 (full results are shown in Additional file 1: Tables S2 and S3). The correlations for glucose, as measured by the mass spectrometer, provide an anchor for interpreting this data – LC-MS glucose had a strong positive correlation with glucose measured by the clinical laboratory (*r* = 0.80, *P* <10^−15^), and a modest negative correlation with serum sodium (*r* = −0.21, *P* = 7.0 × 10^−3^) that did not reach statistical significance, but is well recognized in the clinic (*i.e.* dilutional hyponatremia due to hyperglycemia). Aside from the correlation between the two glucose measures, the strongest correlations identified by this broader examination of uremic metabolites were indeed between long-chain acylcarnitines and lipid traits (total and LDL cholesterol) and blood pressure. Among lipid phenotypes, long-chain acylcarnitines such as oleoylcarnitine were more strongly correlated with total (*r* = −0.45, *P* = 2.6 × 10^−9^) and LDL cholesterol (*r* = −0.43., *P* = 1.7 × 10^−8^) than with HDL cholesterol (*r* = −0.15, *P* = 5.7 × 10^−2^) or triglycerides (*r* = −0.27, *P* = 5.0 × 10^−4^). Similarly, oleoylcarnitine was more strongly correlated with SBP (*r* = −0.38, *P* = 3.4 × 10^−8^) than diastolic blood pressure (*r* = −0.25, *P* = 3.0 × 10^−3^). Long-chain acylcarnitines were not significantly correlated with any non-lipid or non-blood pressure phenotype.

Among the other uremic retention solutes included in our analysis, only kynurenic and anthranilic acid (both tryptophan metabolites downstream of IDO) had more than one significant correlation with a clinical variable (Fig. 6). Kynurenic acid was significantly correlated with diastolic blood pressure (*r* = 0.32, *P* = 5.0 **×** 10^−6^), bicarbonate (*r* = −0.31, *P* = 1.1 **×** 10^−5^), and phosphorous (*r* = 0.38, *P* = 5.7 **×** 10^−8^), whereas anthranilic acid was significantly correlated with alkaline phosphatase (r = 0.29, *P* = 6.3 **×** 10^−5^) and ferritin (*r* = 0.35, *P* = 9.7 **×** 10^−7^), a frequently used marker of inflammation in hemodialysis. Notably, the other metabolites that had a nominal association with ferritin included two other tryptophan metabolites, kynurenic acid (*r* = 0.16, *P* = 2.6 **×** 10^−2^) and quinolinate (*r* = 0.20, *P* = 6.3 **×** 10^−3^), and malondialydehyde (*r* = 0.24, *P* = 9.0 **×** 10^−4^), an oxidative stress marker. These findings reinforce a potential link between the IDO pathway and uremic inflammation. Sorbitol (*r* = −0.34, *P* = 3.4 × 10^−5^), cytidine (r = −0.40, *P* = 1.1 **×** 10^−6^), and uracil (r = −0.42, *P* = 1.7 **×** 10^−7^) were significantly correlated with plasma glucose; urate (*r* = −0.35, *P* = 5.2 **×** 10^−6^) and malondialdehyde (*r* = 0.38, *P* = 5.7 **×** 10^−7^) were significantly correlated with triglycerides; hippurate (*r* = 0.31, *P* = 7.0 **×** 10^−6^) and oxalate (*r* = 0.36, *P* = 1.6 **×** 10^−7^) were significantly correlated with phosphorous; and indoxyl sulfate (*r* = −0.28, *P* = 6.8 **×** 10^−5^) was significantly correlated with bicarbonate. Indoxyl sulfate, which has been found to have several proinflammatory effects in model systems [52], had no correlation with ferritin (nor transferrin saturation, nor white blood cell count), but had a trend for a negative correlation with BNP (*r* = −0.44, *P* = 4.0 **×** 10^−4^).

Although significant work has been devoted to understanding the *in vitro* effects of uremic retention solutes, less is known about how many of these metabolites relate to clinical traits in humans. Thus, in addition to demonstrating the specificity of select long-chain acylcarnitine associations, our examination of uremic metabolites against clinical phenotypes provides a resource for future uremic toxin research.

### Limitations

Our study has several limitations. First, by examining cross-sectional relationships between metabolite levels and various clinical phenotypes, our study is unable to address causation. Instead, by applying parsimonious selection procedures and conservative adjusted significance thresholds, we seek to highlight notable associations for future investigations. Second, study subjects were selected in the context of a case–control study of 1-year cardiovascular mortality, raising the possibility of confounding by case status. However, as noted in the Methods, the statistical significance of the findings are not changed when analyzed stratified by case status. Nevertheless, we acknowledge that our sample is not a random selection of incident dialysis patients, potentially limiting the generalizability of our findings. Third, residual confounding from other sources is likely to influence the associations described, as multivariate adjustments were not pursued. However, because the relationship between various clinical phenotypes and metabolite levels in uremia is unknown, we did not want to obscure novel biological associations by statistical adjustment. Thus, we present these raw associations as a framework for interpreting and appropriately adjusting select findings in future metabolomics studies in ESRD. Finally, our results require replication in an independent sample, ideally selected randomly and including both incident and prevalent dialysis patients.

## Conclusions

In this report, we describe the small molecule alterations that accompany various metabolic phenotypes in ESRD. For phenotypes like diabetes and obesity that have been examined using metabolomics in the general population, our data reinforce the notion that unique pathophysiologic processes arise at ESRD onset. Our finding that long-chain acylcarnitines are strongly and inversely correlated with cholesterol and blood pressure corroborate their potential value as markers of cardiovascular risk in ESRD, whereas the association of tryptophan levels across several phenotypes is of interest given recent studies that assign a functional role for tryptophan metabolism in inflammation and vascular biology. Ultimately, we hope that the breadth of the data presented herein serves as a springboard for investigating mechanisms of uremic toxicity and identifying therapeutic targets to improve the unacceptably high morbidity and mortality attributable to ESRD.

## Additional file

Additional file 1: Table S1. Metabolites measured within the sample set. **Table S2.** Pearson correlation coefficients for select metabolites and available clinical variables in the ArMORR cohort. **Table S3.** *P*-values from Pearson correlations using select metabolites and available clinical variables in the ArMORR cohort.

## Abbreviations

ESRD: End stage renal disease
LDL: Low density lipoprotein
BMI: Body mass index
LC-MS: Liquid chromatography-mass spectrometry
ArMORR cohort: Accelerated Mortality on Renal Replacement cohort
SBP: Systolic blood pressure
PLS-DA: Partial least squares discriminant analysis
VIP: Variable importance in projection
XMP: Xanthosine-5-phosphate
VMA: Vanillylmandelic acid
IDO: Indoleamine-2,3-dioxygenase
BNP: b-type natriuretic peptide
