# Supplementary material for: Cross-sectional examination of metabolites and metabolic phenotypes in uremia
Source: BMC Nephrol. 2015 Jul 7;16:98. doi: 10.1186/s12882-015-0100-y (PMC4491861; doi:10.1186/s12882-015-0100-y)
Supplement: Additional file 1: Table S1. — Metabolites measured within the sample set. Table S2. Pearson correlation coefficients for select metabolites and available clinical variables in the ArMORR cohort. Table S3. P-values from Pearson correlations using select metabolites and available clinical variables in the ArMORR cohort. [file 12882_2015_100_MOESM1_ESM.docx]

**Supplemental Materials**

**Table S1. Metabolites measured within the sample set**

1-methylhistamine

1-methylnicotinamide

2-deoxyadenosine

2-hydroxyglutarate

3-hydroxyanthranilic acid

3-methyladipate; pimelate

3-phosphoglyceric acid (3-PG)

5-aminolevulinic acid

5-hydroxyindoleacetic acid

5-hydroxytryptophan

6-phosphogluconate

acetylcarnitine (2:0 carnitine)

acetylglycine

aconitate

adenine

adenosine

adenosine diphosphate (ADP)

adenosine monophosphate (AMP)

adipate

alanine

allantoin

alpha-glycerophosphate

alpha-glycerophosphocholine

alpha-hydroxybutyric acid

alpha-ketoglutarate

aminoadipic acid (aminoadipate)

anserine

anthranilic acid

arachidonyl carnitine (20:4 carnitine)

arginine

asparagine

aspartate

asymmetric dimethyl-arginine (ADMA)

benzoate

beta-alanine

beta-aminoisobutyric acid (BAIBA)

beta-hydroxybutyric acid (b-OH-butyrate)

betaine

bilirubin

butyrobetaine

butyrylcarnitine (4:0 carnitine)

carnitine

carnosine

cholate

choline

citrate

citrulline

cotinine

creatine

creatinine

cystathionine

cytidine

cytidine diphosphate

cytidine monophosphate

cytosine

decanoylcarnitine (10:0 carnitine)

deoxycytidine monophosphate

dihydroxyacetone phosphate; glyceraldehyde-3-phosphate

dimethylglycine

erythrose-4-phosphate

fructose-1,6-diphosphate; fructose-2,6-diphosphate; glucose-1,6-diphosphate

fructose-1-phosphate; fructose-6-phosphate; glucose-1-phosphate; glucose-6-phosphate

fumarate; maleate; alpha-ketoisovalerate

gamma-aminoisobutyric acid

gentistate

glucose

glucuronate

glutamate

glutamine

glycerol

glycine

glycocholate

glycodeoxycholate-glycochenodeoxycholate (GCDCAs)

guanosine monophosphate (GMP)

heptanoylcarnitine (7:0 carnitine)

hexacosanoylcarnitine (26:0 carnitine)

hexanoylcarnitine (6:0 carnitine)

hippurate

histamine

histidine

hydroxyphenylacetate

hydroxyproline

hyodeoxycholate; ursodeoxycholate; chenodeoxycholate; deoxycholate

hypoxanthine

indole-3-propionate (IPA)

indolelactate

indoxyl sulfate

inosine

inosine monophosphate

inositol

isocitrate

isoleucine

kynurenic acid

kynurenine

lactate

lactose

lauroylcarnitine (12:0 carnitine)

leucine

linoleylcarnitine (18:2 carnitine)

lithocholate

lysine

malate

malondialdehyde

malonylcarnitine (3:0b carnitine)

methionine

methionine sulfoxide

mevalonate 5-phosphate (mevalonate-5P)

myristoylcarnitine (14:0 carnitine)

NG-monomethyl-arginine

niacinamide

nonanoylcarnitine (9:0 carnitine)

octanoylcarnitine (8:0 carnitine)

oleoylcarnitine (18:1 carnitine)

ornithine

orotate

oxalate

palmitoylcarnitine (16:1 carnitine)

pantothenate

phenylalanine

phosphocreatine (p-creatine)

phosphoenolpyruvic acid (PEP)

pipecolic acid

proline

propionate

propionylcarnitine (3:0 carnitine)

putrescine

pyridoxate

pyroglutamic acid

pyruvate

quinolinate

ribose-5-phosphate; ribulose-5-phosphate (ribose-5-P-rib)

salicylurate

sarcosine

serine

serotonin

sorbitol

spermidine

stearoylcarnitine (18:0 carnitine)

suberate

succinate

sucrose

symmetric dimethyl-arginine (SDMA)

taurine

taurocholate

taurodeoxycholate; taurochenodeoxycholate

thiamine

threonine

thymidine

thymine

thyroxine

trimethylamine-N-oxide

tryptophan

tyrosine

uracil

urate

ureidopropionic acid

uridine

uridine diphosphate

uridine diphosphate glucose; uridine diphosphate galactose

uridine monophosphate

valerylcarnitine (5:0 carnitine)

valine

vanillylmandelic acid (VMA)

xanthine

xanthosine

xanthosine-5-phosphate (XMP)

**Table S2. Pearson correlation coefficients for select metabolites and available clinical variables in the ArMORR cohort**

|  | **Age** | **BMI** | **URR** | **SBP** | **DBP** | **Cholesterol** | **LDL** | **HDL** | **Triglycerides** | **BNP** | **Troponin** | **Glucose** | **Albumin** |
| --- | --- | --- | --- | --- | --- | --- | --- | --- | --- | --- | --- | --- | --- |
| **Sorbitol** | -0.20 | 0.03 | -0.08 | -0.10 | 0.00 | -0.14 | -0.14 | -0.09 | -0.03 | -0.02 | 0.07 | -0.34 | -0.11 |
| **Glucose** | 0.08 | 0.03 | 0.03 | 0.01 | -0.02 | -0.05 | -0.03 | 0.16 | -0.12 | -0.02 | -0.02 | 0.80 | 0.04 |
| **Cytidine** | 0.00 | -0.02 | -0.10 | -0.20 | -0.15 | -0.13 | -0.12 | -0.16 | -0.03 | 0.08 | 0.02 | -0.40 | -0.11 |
| **Uracil** | -0.05 | -0.01 | -0.03 | -0.14 | -0.11 | 0.10 | 0.09 | -0.14 | 0.14 | 0.14 | 0.12 | -0.42 | -0.10 |
| **Uridine** | 0.00 | 0.00 | -0.12 | -0.12 | -0.10 | -0.05 | -0.03 | -0.19 | 0.06 | -0.03 | -0.07 | -0.32 | 0.02 |
| **Xanthine** | 0.08 | -0.03 | 0.04 | -0.14 | -0.16 | -0.09 | -0.09 | -0.12 | 0.08 | 0.10 | -0.01 | -0.11 | 0.06 |
| **Xanthosine** | -0.05 | 0.04 | -0.06 | -0.02 | -0.05 | 0.01 | 0.05 | -0.15 | 0.11 | -0.13 | 0.05 | -0.08 | -0.03 |
| **Urate** | 0.05 | 0.08 | -0.01 | -0.06 | -0.08 | -0.18 | -0.13 | 0.07 | -0.35 | 0.00 | 0.00 | 0.13 | -0.01 |
| **Kynurenine** | 0.08 | 0.14 | 0.06 | -0.06 | 0.05 | -0.22 | -0.16 | -0.21 | 0.04 | 0.04 | -0.16 | -0.04 | 0.28 |
| **Orotate** | 0.04 | -0.01 | 0.12 | -0.21 | -0.21 | -0.02 | 0.00 | -0.20 | 0.10 | 0.07 | -0.01 | -0.01 | 0.01 |
| **ADMA** | -0.04 | 0.00 | 0.07 | -0.20 | -0.09 | 0.00 | 0.00 | 0.02 | 0.00 | 0.08 | 0.25 | 0.11 | 0.09 |
| **Indoxyl Sulfate** | -0.09 | 0.05 | -0.05 | 0.16 | 0.15 | 0.12 | 0.18 | -0.06 | 0.03 | -0.44 | -0.14 | -0.03 | 0.23 |
| **TMAO** | -0.07 | 0.05 | -0.06 | 0.08 | 0.07 | 0.02 | 0.05 | -0.01 | 0.00 | -0.23 | -0.18 | -0.02 | 0.11 |
| **Hippurate** | -0.15 | 0.02 | -0.14 | 0.20 | 0.21 | 0.11 | 0.18 | 0.11 | 0.00 | 0.01 | -0.11 | 0.01 | 0.03 |
| **Kynurenic acid** | -0.23 | 0.09 | -0.19 | 0.26 | 0.32 | 0.10 | 0.20 | -0.04 | 0.05 | 0.06 | 0.03 | -0.07 | 0.04 |
| **Malondialdehyde** | 0.04 | -0.01 | 0.12 | 0.09 | 0.02 | 0.29 | 0.17 | -0.09 | 0.38 | -0.20 | -0.05 | 0.06 | 0.05 |
| **Quinolinate** | -0.01 | 0.09 | 0.02 | -0.08 | 0.00 | 0.00 | 0.03 | -0.28 | 0.16 | 0.20 | 0.06 | -0.14 | 0.01 |
| **Anthranilic acid** | 0.06 | -0.05 | 0.08 | 0.00 | 0.02 | 0.18 | 0.17 | -0.10 | 0.15 | 0.06 | 0.03 | -0.13 | -0.09 |
| **Oxalate** | -0.02 | -0.02 | -0.05 | -0.07 | -0.12 | 0.06 | 0.05 | 0.09 | 0.08 | 0.20 | 0.03 | 0.02 | -0.04 |
| **Thymine** | -0.07 | 0.03 | -0.11 | -0.13 | -0.10 | 0.04 | 0.02 | -0.04 | 0.01 | 0.25 | 0.08 | -0.04 | -0.20 |
| **SDMA** | -0.15 | -0.05 | 0.01 | -0.01 | 0.11 | 0.07 | 0.08 | -0.02 | -0.11 | 0.07 | 0.29 | -0.13 | -0.16 |
| **Creatine** | 0.01 | 0.01 | 0.22 | -0.05 | -0.12 | 0.15 | 0.06 | 0.19 | 0.02 | 0.02 | 0.22 | 0.16 | -0.24 |
| **Dimethylglycine** | 0.00 | -0.07 | 0.11 | 0.01 | -0.07 | 0.13 | 0.17 | -0.08 | 0.16 | 0.01 | -0.09 | 0.03 | -0.05 |
| **Hypoxanthine** | -0.13 | 0.04 | -0.08 | -0.01 | 0.00 | 0.16 | 0.20 | -0.08 | 0.08 | -0.02 | 0.08 | -0.09 | -0.05 |
| **Niacinamide** | -0.13 | 0.16 | 0.05 | 0.08 | 0.13 | 0.16 | 0.15 | -0.02 | 0.16 | 0.07 | 0.05 | -0.10 | 0.02 |
| **16:0 carnitine** | 0.07 | 0.06 | 0.08 | -0.35 | -0.24 | -0.24 | -0.25 | -0.13 | -0.08 | 0.20 | 0.29 | 0.00 | 0.13 |
| **18:0 carnitine** | 0.10 | 0.03 | -0.02 | -0.35 | -0.23 | -0.36 | -0.33 | -0.16 | -0.25 | 0.24 | 0.24 | 0.01 | 0.12 |
| **18:1carnitine** | 0.13 | 0.08 | 0.05 | -0.38 | -0.25 | -0.45 | -0.43 | -0.15 | -0.27 | 0.14 | 0.23 | -0.01 | 0.10 |
| **18:2 carnitine** | 0.11 | 0.09 | 0.03 | -0.37 | -0.27 | -0.46 | -0.44 | -0.12 | -0.31 | 0.05 | 0.09 | -0.03 | 0.08 |

**Table S2 (continued)**

|  | **TSAT** | **Ferritin** | **Alk Phos** | **Sodium** | **Potassium** | **Bicarbonate** | **Calcium** | **Phosphate** | **PTH** | **Hemoglobin** | **WBC** |
| --- | --- | --- | --- | --- | --- | --- | --- | --- | --- | --- | --- |
| **Sorbitol** | -0.07 | -0.05 | -0.01 | -0.08 | 0.07 | 0.08 | 0.03 | 0.02 | 0.09 | -0.02 | 0.06 |
| **Glucose** | -0.05 | -0.12 | 0.08 | -0.21 | 0.03 | -0.10 | -0.06 | 0.08 | -0.06 | -0.20 | -0.23 |
| **Cytidine** | 0.00 | 0.09 | -0.06 | 0.11 | -0.20 | 0.25 | 0.04 | -0.10 | -0.03 | 0.11 | 0.17 |
| **Uracil** | -0.01 | 0.09 | -0.02 | 0.04 | -0.03 | 0.06 | 0.08 | 0.08 | 0.07 | 0.16 | 0.20 |
| **Uridine** | 0.05 | 0.14 | 0.03 | -0.01 | -0.03 | 0.22 | 0.17 | -0.17 | -0.05 | 0.20 | -0.04 |
| **Xanthine** | 0.17 | 0.12 | 0.06 | -0.03 | -0.03 | 0.10 | 0.19 | -0.13 | -0.09 | 0.17 | 0.19 |
| **Xanthosine** | 0.13 | 0.08 | -0.06 | -0.07 | 0.03 | 0.09 | 0.14 | -0.01 | -0.01 | 0.19 | 0.08 |
| **Urate** | -0.11 | -0.14 | -0.02 | 0.11 | -0.04 | -0.11 | -0.14 | 0.12 | 0.04 | -0.22 | 0.06 |
| **Kynurenine** | -0.12 | 0.00 | 0.01 | -0.05 | 0.00 | 0.00 | 0.11 | -0.06 | -0.11 | 0.02 | -0.12 |
| **Orotate** | -0.05 | 0.05 | -0.02 | 0.11 | -0.07 | 0.20 | 0.18 | 0.01 | 0.05 | -0.02 | -0.08 |
| **ADMA** | 0.02 | -0.08 | -0.08 | -0.11 | 0.10 | 0.13 | 0.07 | -0.07 | -0.04 | 0.01 | -0.11 |
| **Indoxyl Sulfate** | -0.06 | 0.01 | -0.04 | -0.01 | 0.15 | -0.28 | -0.11 | 0.18 | 0.04 | -0.12 | 0.01 |
| **TMAO** | 0.04 | 0.01 | -0.03 | 0.09 | 0.17 | -0.09 | -0.01 | 0.11 | -0.05 | -0.08 | 0.02 |
| **Hippurate** | -0.12 | 0.06 | -0.08 | -0.05 | 0.20 | -0.20 | -0.19 | 0.31 | 0.10 | -0.13 | -0.12 |
| **Kynurenic acid** | 0.05 | 0.16 | 0.01 | 0.01 | 0.16 | -0.31 | -0.15 | 0.38 | 0.11 | -0.04 | 0.02 |
| **Malondialdehyde** | 0.21 | 0.24 | 0.03 | -0.05 | -0.02 | 0.05 | 0.20 | -0.03 | -0.07 | 0.21 | 0.02 |
| **Quinolinate** | -0.02 | 0.20 | 0.15 | -0.10 | -0.02 | -0.11 | 0.02 | 0.14 | -0.01 | -0.08 | 0.00 |
| **Anthranilic acid** | 0.07 | 0.35 | 0.29 | -0.07 | 0.01 | 0.02 | 0.09 | 0.07 | -0.05 | 0.10 | 0.06 |
| **Oxalate** | 0.11 | 0.14 | -0.01 | -0.09 | 0.20 | -0.19 | -0.14 | 0.36 | 0.08 | -0.09 | -0.01 |
| **Thymine** | -0.03 | 0.07 | 0.01 | -0.05 | -0.06 | 0.18 | -0.03 | 0.07 | -0.02 | 0.12 | 0.07 |
| **SDMA** | 0.03 | 0.06 | 0.15 | -0.05 | 0.04 | -0.04 | 0.03 | 0.15 | 0.12 | 0.00 | 0.03 |
| **Creatine** | 0.06 | 0.07 | -0.01 | -0.15 | 0.04 | 0.01 | -0.06 | 0.11 | -0.04 | -0.03 | 0.08 |
| **Dimethylglycine** | -0.22 | -0.04 | -0.18 | 0.03 | -0.04 | -0.02 | -0.06 | 0.10 | 0.10 | 0.03 | 0.09 |
| **Hypoxanthine** | 0.07 | 0.07 | -0.14 | 0.06 | 0.11 | -0.06 | 0.11 | 0.23 | 0.01 | 0.25 | -0.03 |
| **Niacinamide** | -0.03 | 0.09 | -0.01 | 0.02 | -0.04 | -0.04 | 0.03 | 0.09 | 0.02 | 0.20 | 0.01 |
| **16:0 carnitine** | -0.10 | -0.02 | -0.02 | -0.06 | -0.08 | 0.15 | 0.10 | -0.11 | -0.07 | 0.22 | -0.16 |
| **18:0 carnitine** | -0.13 | 0.00 | -0.06 | 0.05 | -0.15 | 0.20 | 0.03 | -0.15 | -0.10 | 0.20 | -0.18 |
| **18:1carnitine** | -0.13 | -0.06 | -0.04 | -0.04 | -0.13 | 0.22 | 0.07 | -0.15 | -0.08 | 0.15 | -0.16 |
| **18:2 carnitine** | -0.19 | -0.14 | -0.08 | -0.06 | -0.07 | 0.21 | 0.03 | -0.06 | -0.01 | 0.09 | -0.18 |

Abbreviations: BMI, body mass index; URR, urea reduction ratio; SBP, systolic blood pressure; DBP, diastolic blood pressure; Cholesterol, total cholesterol; LDL, low-density lipoprotein cholesterol; HDL, high-density lipoprotein cholesterol; BNP, brain natriuretic peptide; TSAT, transferrin saturation; Alk Phos, alkaline phosphatase; PTH, parathyroid hormone; WBC, white blood cell count.

**Table S3. *P*-values from Pearson correlations using select metabolites and available clinical variables in the ArMORR cohort**

|  | **Age** | **BMI** | **URR** | **SBP** | **DBP** | **Cholesterol** | **LDL** | **HDL** | **Triglycerides** | **BNP** | **Troponin** | **Glucose** |
| --- | --- | --- | --- | --- | --- | --- | --- | --- | --- | --- | --- | --- |
| **Sorbitol** | 5.2E-3 | 6.4E-1 | 3.5E-1 | 1.5E-1 | 9.6E-1 | 7.7E-2 | 6.8E-2 | 2.4E-1 | 7.1E-1 | 8.8E-1 | 5.9E-1 | 3.4E-5* |
| **Glucose** | 2.8E-1 | 6.9E-1 | 7.4E-1 | 8.6E-1 | 7.7E-1 | 5.4E-1 | 6.8E-1 | 4.3E-2 | 1.4E-1 | 8.8E-1 | 8.6E-1 | 10E-15* |
| **Cytidine** | 9.6E-1 | 7.7E-1 | 2.1E-1 | 4.4E-3 | 3.3E-2 | 1.1E-1 | 1.2E-1 | 4.1E-2 | 7.1E-1 | 5.3E-1 | 8.5E-1 | 1.1E-6* |
| **Uracil** | 5.2E-1 | 8.9E-1 | 7.5E-1 | 4.8E-2 | 1.1E-1 | 2.0E-1 | 2.6E-1 | 7.5E-2 | 6.7E-2 | 2.9E-1 | 3.5E-1 | 1.8E-7* |
| **Uridine** | 9.5E-1 | 9.8E-1 | 1.4E-1 | 8.4E-2 | 1.5E-1 | 5.6E-1 | 6.7E-1 | 1.7E-2 | 4.2E-1 | 8.3E-1 | 6.0E-1 | 1.1E-4 |
| **Xanthine** | 2.6E-1 | 7.0E-1 | 6.2E-1 | 5.3E-2 | 2.5E-2 | 2.4E-1 | 2.5E-1 | 1.2E-1 | 3.1E-1 | 4.5E-1 | 9.3E-1 | 1.8E-1 |
| **Xanthosine** | 4.8E-1 | 5.9E-1 | 4.7E-1 | 7.9E-1 | 4.9E-1 | 8.5E-1 | 5.4E-1 | 5.7E-2 | 1.6E-1 | 3.4E-1 | 7.1E-1 | 3.5E-1 |
| **Urate** | 5.0E-1 | 2.9E-1 | 9.3E-1 | 4.4E-1 | 2.9E-1 | 1.9E-2 | 1.0E-1 | 3.6E-1 | 5.2E-6* | 9.8E-1 | 9.8E-1 | 1.4E-1 |
| **Kynurenine** | 2.8E-1 | 5.2E-2 | 4.4E-1 | 4.3E-1 | 5.1E-1 | 5.4E-3 | 4.0E-2 | 7.0E-3 | 6.1E-1 | 7.8E-1 | 2.4E-1 | 6.3E-1 |
| **Orotate** | 5.6E-1 | 8.7E-1 | 1.4E-1 | 2.9E-3 | 3.1E-3 | 7.7E-1 | 9.5E-1 | 1.3E-2 | 2.2E-1 | 6.0E-1 | 9.6E-1 | 8.6E-1 |
| **ADMA** | 5.8E-1 | 9.6E-1 | 3.7E-1 | 3.9E-3 | 1.9E-1 | 9.5E-1 | 9.7E-1 | 8.0E-1 | 9.6E-1 | 5.4E-1 | 5.5E-2 | 1.9E-1 |
| **Indoxyl Sulfate** | 2.3E-1 | 4.7E-1 | 5.6E-1 | 2.4E-2 | 3.7E-2 | 1.3E-1 | 2.0E-2 | 4.3E-1 | 7.3E-1 | 4.3E-4 | 2.9E-1 | 7.0E-1 |
| **TMAO** | 3.5E-1 | 4.5E-1 | 4.4E-1 | 2.6E-1 | 3.5E-1 | 8.1E-1 | 5.5E-1 | 8.9E-1 | 9.6E-1 | 7.8E-2 | 1.7E-1 | 8.4E-1 |
| **Hippurate** | 3.6E-2 | 8.0E-1 | 7.6E-2 | 4.6E-3 | 2.6E-3 | 1.7E-1 | 2.5E-2 | 1.8E-1 | 9.7E-1 | 9.5E-1 | 4.1E-1 | 8.9E-1 |
| **Kynurenic acid** | 1.2E-3 | 1.9E-1 | 1.9E-2 | 1.8E-4 | 5.0E-6* | 2.0E-1 | 1.0E-2 | 6.4E-1 | 5.4E-1 | 6.6E-1 | 8.5E-1 | 3.8E-1 |
| **Malondialdehyde** | 5.9E-1 | 9.3E-1 | 1.5E-1 | 1.9E-1 | 7.7E-1 | 2.4E-4 | 2.9E-2 | 2.6E-1 | 5.7E-7* | 1.3E-1 | 7.2E-1 | 4.6E-1 |
| **Quinolinate** | 8.5E-1 | 2.0E-1 | 8.2E-1 | 2.8E-1 | 9.9E-1 | 9.8E-1 | 7.4E-1 | 4.0E-4 | 4.6E-2 | 1.3E-1 | 6.3E-1 | 9.6E-2 |
| **Anthranilic acid** | 4.1E-1 | 5.2E-1 | 3.4E-1 | 9.6E-1 | 7.8E-1 | 2.2E-2 | 3.0E-2 | 2.2E-1 | 6.3E-2 | 6.3E-1 | 8.5E-1 | 1.3E-1 |
| **Oxalate** | 7.8E-1 | 8.2E-1 | 5.5E-1 | 3.6E-1 | 8.4E-2 | 4.5E-1 | 5.0E-1 | 2.5E-1 | 2.9E-1 | 1.2E-1 | 8.1E-1 | 8.4E-1 |
| **Thymine** | 3.4E-1 | 7.2E-1 | 1.7E-1 | 6.4E-2 | 1.8E-1 | 6.3E-1 | 7.7E-1 | 5.8E-1 | 8.6E-1 | 5.9E-2 | 5.4E-1 | 6.5E-1 |
| **SDMA** | 3.3E-2 | 5.0E-1 | 9.4E-1 | 8.6E-1 | 1.3E-1 | 3.6E-1 | 2.9E-1 | 8.2E-1 | 1.6E-1 | 6.1E-1 | 2.8E-2 | 1.2E-1 |
| **Creatine** | 8.8E-1 | 9.4E-1 | 5.0E-3 | 4.4E-1 | 9.0E-2 | 5.4E-2 | 4.7E-1 | 1.8E-2 | 7.9E-1 | 8.7E-1 | 9.1E-2 | 5.3E-2 |
| **Dimethylglycine** | 9.7E-1 | 3.1E-1 | 1.6E-1 | 8.8E-1 | 3.3E-1 | 9.7E-2 | 3.0E-2 | 3.0E-1 | 4.8E-2 | 9.5E-1 | 4.8E-1 | 7.1E-1 |
| **Hypoxanthine** | 6.6E-2 | 6.2E-1 | 3.2E-1 | 9.2E-1 | 9.7E-1 | 4.1E-2 | 9.4E-3 | 2.9E-1 | 2.9E-1 | 8.6E-1 | 5.3E-1 | 3.1E-1 |
| **Niacinamide** | 6.2E-2 | 2.1E-2 | 4.9E-1 | 2.5E-1 | 6.7E-2 | 4.5E-2 | 5.2E-2 | 7.9E-1 | 5.0E-2 | 6.1E-1 | 7.1E-1 | 2.6E-1 |
| **16:0 carnitine** | 3.2E-1 | 3.8E-1 | 3.3E-1 | 5.0E-7* | 7.4E-4 | 2.6E-3 | 1.4E-3 | 1.0E-1 | 2.9E-1 | 1.3E-1 | 2.5E-2 | 9.9E-1 |
| **18:0 carnitine** | 1.4E-1 | 6.8E-1 | 7.9E-1 | 4.5E-7* | 1.2E-3 | 2.2E-6* | 2.4E-5* | 4.2E-2 | 1.2E-3 | 6.4E-2 | 6.4E-2 | 9.4E-1 |
| **18:1carnitine** | 6.0E-2 | 2.9E-1 | 5.7E-1 | 3.4E-8* | 3.0E-4 | 2.6E-9* | 1.7E-8* | 5.7E-2 | 5.1E-4 | 2.8E-1 | 7.9E-2 | 8.8E-1 |
| **18:2 carnitine** | 1.4E-1 | 2.1E-1 | 6.8E-1 | 9.6E-8* | 1.0E-4 | 1.2E-9* | 4.8E-9* | 1.3E-1 | 6.6E-5* | 7.0E-1 | 5.1E-1 | 7.5E-1 |

**Table S3 (continued)**

|  | **Albumin** | **TSAT** | **Ferritin** | **Alk Phos** | **Sodium** | **Potassium** | **Bicarbonate** | **Calcium** | **Phosphate** | **PTH** | **Hemoglobin** | **WBC** |
| --- | --- | --- | --- | --- | --- | --- | --- | --- | --- | --- | --- | --- |
| **Sorbitol** | 1.1E-1 | 3.6E-1 | 5.1E-1 | 8.8E-1 | 3.1E-1 | 3.2E-1 | 3.0E-1 | 6.6E-1 | 7.7E-1 | 2.4E-1 | 7.8E-1 | 3.9E-1 |
| **Glucose** | 5.9E-1 | 5.2E-1 | 9.2E-2 | 2.8E-1 | 7.0E-3 | 6.4E-1 | 1.6E-1 | 4.1E-1 | 2.5E-1 | 4.5E-1 | 4.4E-3 | 1.6E-3 |
| **Cytidine** | 1.2E-1 | 9.5E-1 | 2.2E-1 | 4.5E-1 | 1.9E-1 | 5.0E-3 | 4.4E-4 | 5.7E-1 | 1.7E-1 | 7.2E-1 | 1.2E-1 | 1.6E-2 |
| **Uracil** | 1.6E-1 | 8.5E-1 | 2.1E-1 | 8.1E-1 | 6.1E-1 | 7.2E-1 | 4.1E-1 | 2.5E-1 | 2.5E-1 | 3.4E-1 | 2.1E-2 | 5.0E-3 |
| **Uridine** | 8.1E-1 | 4.9E-1 | 4.9E-2 | 6.8E-1 | 9.1E-1 | 6.8E-1 | 2.4E-3 | 1.7E-2 | 1.8E-2 | 5.4E-1 | 5.6E-3 | 5.4E-1 |
| **Xanthine** | 4.2E-1 | 1.7E-2 | 8.9E-2 | 4.2E-1 | 7.4E-1 | 6.7E-1 | 1.6E-1 | 6.1E-3 | 6.9E-2 | 2.2E-1 | 1.5E-2 | 7.9E-3 |
| **Xanthosine** | 6.2E-1 | 7.1E-2 | 3.0E-1 | 4.2E-1 | 3.6E-1 | 6.6E-1 | 2.3E-1 | 4.9E-2 | 8.8E-1 | 9.4E-1 | 8.4E-3 | 2.5E-1 |
| **Urate** | 9.4E-1 | 1.3E-1 | 5.7E-2 | 7.5E-1 | 1.6E-1 | 5.9E-1 | 1.1E-1 | 4.6E-2 | 1.0E-1 | 6.3E-1 | 2.1E-3 | 3.9E-1 |
| **Kynurenine** | 8.1E-5 | 1.0E-1 | 9.9E-1 | 8.7E-1 | 5.4E-1 | 9.8E-1 | 9.5E-1 | 1.4E-1 | 4.2E-1 | 1.3E-1 | 7.8E-1 | 9.2E-2 |
| **Orotate** | 9.1E-1 | 5.1E-1 | 5.0E-1 | 7.5E-1 | 1.6E-1 | 3.2E-1 | 5.8E-3 | 9.8E-3 | 8.7E-1 | 4.8E-1 | 8.3E-1 | 2.8E-1 |
| **ADMA** | 1.9E-1 | 7.4E-1 | 2.6E-1 | 3.1E-1 | 1.7E-1 | 1.9E-1 | 6.5E-2 | 3.2E-1 | 3.1E-1 | 6.0E-1 | 8.6E-1 | 1.2E-1 |
| **Indoxyl Sulfate** | 1.3E-3 | 4.0E-1 | 8.8E-1 | 5.5E-1 | 9.3E-1 | 3.7E-2 | 6.8E-5* | 1.3E-1 | 9.8E-3 | 5.7E-1 | 1.0E-1 | 9.4E-1 |
| **TMAO** | 1.1E-1 | 5.7E-1 | 8.4E-1 | 7.2E-1 | 2.7E-1 | 1.5E-2 | 2.2E-1 | 9.0E-1 | 1.4E-1 | 4.6E-1 | 2.8E-1 | 8.3E-1 |
| **Hippurate** | 6.3E-1 | 1.1E-1 | 4.0E-1 | 2.9E-1 | 5.1E-1 | 6.1E-3 | 5.3E-3 | 6.5E-3 | 7.0E-6* | 1.6E-1 | 7.2E-2 | 9.0E-2 |
| **Kynurenic acid** | 5.8E-1 | 4.9E-1 | 2.6E-2 | 9.3E-1 | 8.7E-1 | 3.1E-2 | 1.1E-5* | 3.5E-2 | 5.7E-8* | 1.3E-1 | 6.0E-1 | 7.9E-1 |
| **Malondialdehyde** | 4.9E-1 | 4.2E-3 | 9.2E-4 | 6.5E-1 | 5.2E-1 | 8.2E-1 | 4.7E-1 | 4.9E-3 | 6.9E-1 | 3.3E-1 | 3.7E-3 | 7.6E-1 |
| **Quinolinate** | 8.7E-1 | 7.8E-1 | 6.3E-3 | 4.2E-2 | 2.0E-1 | 7.3E-1 | 1.4E-1 | 7.3E-1 | 5.9E-2 | 8.5E-1 | 2.8E-1 | 9.9E-1 |
| **Anthranilic acid** | 2.1E-1 | 3.6E-1 | 9.7E-7* | 6.3E-5* | 4.2E-1 | 9.1E-1 | 7.4E-1 | 2.2E-1 | 3.4E-1 | 4.9E-1 | 1.6E-1 | 4.4E-1 |
| **Oxalate** | 5.5E-1 | 1.2E-1 | 5.3E-2 | 8.9E-1 | 2.9E-1 | 4.7E-3 | 7.1E-3 | 5.2E-2 | 1.6E-7* | 2.7E-1 | 2.0E-1 | 8.5E-1 |
| **Thymine** | 4.8E-3 | 6.7E-1 | 3.3E-1 | 9.0E-1 | 5.4E-1 | 4.1E-1 | 1.3E-2 | 6.9E-1 | 3.4E-1 | 8.4E-1 | 9.0E-2 | 3.3E-1 |
| **SDMA** | 2.6E-2 | 6.7E-1 | 4.0E-1 | 3.7E-2 | 5.7E-1 | 6.2E-1 | 6.2E-1 | 7.0E-1 | 3.1E-2 | 9.4E-2 | 9.7E-1 | 7.2E-1 |
| **Creatine** | 7.5E-4 | 4.3E-1 | 3.1E-1 | 8.8E-1 | 6.6E-2 | 5.9E-1 | 8.6E-1 | 4.1E-1 | 1.3E-1 | 5.7E-1 | 6.9E-1 | 2.5E-1 |
| **Dimethylglycine** | 5.1E-1 | 2.3E-3 | 6.0E-1 | 1.6E-2 | 7.3E-1 | 5.8E-1 | 7.7E-1 | 4.1E-1 | 1.6E-1 | 1.8E-1 | 6.7E-1 | 2.0E-1 |
| **Hypoxanthine** | 5.2E-1 | 3.7E-1 | 3.3E-1 | 6.0E-2 | 4.5E-1 | 1.3E-1 | 4.2E-1 | 1.2E-1 | 1.1E-3 | 8.9E-1 | 3.8E-4 | 7.2E-1 |
| **Niacinamide** | 7.8E-1 | 6.7E-1 | 2.0E-1 | 8.7E-1 | 8.5E-1 | 5.9E-1 | 6.1E-1 | 6.9E-1 | 2.0E-1 | 7.6E-1 | 5.7E-3 | 9.0E-1 |
| **16:0 carnitine** | 6.7E-2 | 1.7E-1 | 7.4E-1 | 7.4E-1 | 4.6E-1 | 2.8E-1 | 3.6E-2 | 1.6E-1 | 1.3E-1 | 3.8E-1 | 1.8E-3 | 2.8E-2 |
| **18:0 carnitine** | 9.0E-2 | 7.2E-2 | 9.5E-1 | 4.5E-1 | 5.4E-1 | 4.0E-2 | 4.2E-3 | 6.4E-1 | 3.0E-2 | 2.0E-1 | 4.4E-3 | 1.2E-2 |
| **18:1carnitine** | 1.7E-1 | 6.6E-2 | 4.4E-1 | 6.1E-1 | 6.2E-1 | 6.8E-2 | 1.7E-3 | 3.4E-1 | 3.0E-2 | 3.0E-1 | 3.2E-2 | 2.7E-2 |
| **18:2 carnitine** | 2.6E-1 | 7.4E-3 | 6.4E-2 | 2.9E-1 | 4.6E-1 | 3.6E-1 | 3.0E-3 | 7.0E-1 | 3.9E-1 | 9.2E-1 | 1.9E-1 | 1.4E-2 |

Abbreviations: BMI, body mass index; URR, urea reduction ratio; SBP, systolic blood pressure; DBP, diastolic blood pressure; Cholesterol, total cholesterol; LDL, low-density lipoprotein cholesterol; HDL, high-density lipoprotein cholesterol; BNP, brain natriuretic peptide; TSAT, transferrin saturation; Alk Phos, alkaline phosphatase; PTH, parathyroid hormone; WBC, white blood cell count.

* Significant at the Bonferroni corrected value of *P <* 7.4 x 10^-5^.
